# Diet in Disease

**Published:** 1892-11-05

**Authors:** Ernest Hart

**Affiliations:** Bachelier-ès-Sciences-es-Lettres (rèstreint), formerly Student of the Faculty of Medicine of Paris, and of the London School of Medicine for Women


### Diet in Disease.

By Mrs. Ernest Hart, Bachelier-ès-Sciences-es-Lettres (rèstreint), formerly Student of the Faculty of Medicine of Paris, and of the London School of Medicine for Women.

### V.—RESTORATIVES.

### Tea.

The restorative properties of tea are well known and universally aamitted. To partake of "the cup that cheers but not inebriates" has become a national habit, and is indulged in equally by the richest and the poorest.

**How Tea is Prepared.**—Tea consists of the prepared leaves of a small shrub-like plant resembling a camellia. It is cultivated in China, Japan, India, and Ceylon. The leaves are gathered by hand three times in the year; the young and tender shoots make the finest teas. The peculiar astringent quality and aromatic flavour of tea, as we know it, are developed in the processes of drying and roasting. Green tea is prepared from the young leaves which almost immediately after being gathered are cast into shallow pans and roasted over a brisk wood fire. After being thus treated for about five minutes, they are removed thrown upon a table, and rolled with the hands. They are again thrown into the pan and are well shaken about over the fire for an hour and a half, till they are thoroughly dried, when the pale green colour characteristic of green tea is fixed. These slightly roasted and delicate green teas are highly appreciated by the Chinese, the Japanese, and the Russians, but are scarcely used in England except by tea connoisseurs. Many of the teas known in England as "green teas" are not of this fine variety, but are the coarser teas faced or coloured with Prussian blue or indigo. These are, however, little used now. In the preparation of black tea the leaves, after being plucked and brought in from the plantation, are allowed to lie in heaps for about twelve hours. They are then tossed into the air, and patted with the hand by the workmen until they are soft and flaccid; again thrown into heaps, and allowed to remain for some time. They are then rolled into balls, and the sap is squeezed out by the hands of the workmen, the leaves receiving at the same time, a twist. They are then roasted and rolled, in the same way as green tea. After this they are laid out on sieves, and exposed out of doors for three hours to a sunny air. The leaves are again roasted and rolled a second time, and this process is repeated, with slight alterations, three, four, and even five times. The tea leaves are now perfectly dry, of a fine black colour, crisply rolled, and have the appearance we know so well. There is no doubt that fermentative changes take place in the preparation of black tea, which considerably alter the chemical character of the leaf.

**The Constituents of Tea.**—Tea contains three active principles to which it owes its peculiar properties and characteristics. These are *theine*, a crystallisable alkaloid, to which is due its stimulating and restorative properties; *tannin*, whence it derives its astringency and a good deal of what is popularly known as "strength"; and a *volatile oil*, to which it owes its aroma. There is probably also a bitter principle, which has not yet been separated. It is less soluble than tannin, is extracted from the leaves and passes into the tea after a long infusion or stewing. Both theine and tannin are soluble in boiling water. The theine is in combination with tannic acid, and the theine and tannin are together dissolved out of the leaves into the tea. There is a popular impression that by a very short infusion of only two or three minutes, theine can be obtained from tea, without the tannin. This is a mistake. Tannin, which is very soluble, is always dissolved out with the theine. Some teas, however, continue to give off tannin after the chief part of the theine has been dissolved out. These are chiefly the Indian teas, which have been highly fired and much fermented in the process of preparation.

The following table taken from a number of analyses recently made by the late Professor Dittmar, show clearly the effect of allowing the water to stand on the tea leaves five minutes and ten minutes respectively, and the varying amount of theine and tannin given off by China, Ceylon, and Indian teas:—

| Five Minutes Infusion. | | | Ten Minutes Infusion. | | |
|---|---|---|---|---|---|
| | Theine. | Tannin. | | Theine. | Tannin. |
| China ... | 2·58 ... | 3·06 | China ... | 2·79 ... | 3·78 |
| Ceylon ... | 3·15 ... | 5·87 | Ceylon ... | 3·29 ... | 7·30 |
| Indian ... | 3·63 ... | 6·77 | Indian ... | 3·73 ... | 8 09 |

From this it will be seen that though Indian tea contains 25 per cent. more theine than China tea, it also contains 100 per cent. more tannin. Indian teas are much more widely used in England than China teas, and the "strong syrupy teas," advertised as of good value, and so largely consumed by the working classes, are as a rule, blends of various Indian teas rich in tannin and astringent matters. It thus obviously becomes a matter of great, and even of national importance, considering how extensively and continuously tea is drunk, to ascertain the physiological effects of its principal constituents—theine and tannin.

**The Physiological Effects of Tea.**—Universal experience teaches us that tea exhilarates without intoxicating, stimulates the circulation, excites the brain to increased activity, promotes wakefulness, and banishes the sense of weariness. It also deadens the sensation of hunger, and increases the power of fasting. It will cool the body when hot, and warm it when cold. In tropical countries it has been found to be a most valuable restorative when taken by soldiers on long and fatiguing marches. Lord Wolseley, who is a great advocate for tea as a beverage on which to do hard work, gave orders that the water-bottles of the soldiers whom he led on the two famous and exhausting expeditions of the Red River, and up the Nile to Khartoum, should be filled with cold tea; and he is convinced that, whereas alcohol induces fatigue, tea will give the power to endure and overcome it.

**The exhilarating and the staying powers of Tea** are due respectively to the theine and tannin contained in it; the theine exhilarates the nervous system, the tannin stays hunger. This latter point has been made clear by the interesting and valuable experiments of Sir William Roberts, who has shown that tannin, taken even in very small quantities, has an inhibitory or slowing influence on the digestion of food in the stomach. He found that this took place with tea made at the ordinary strength, and taken in the usual amount, with food; the digestion being still longer delayed if a large amount of fluid tea was drunk. He could not, however, detect any appreciable difference between the inhibitory effect of tea infused for two or three minutes and tea infused for fifteen or thirty minutes In fact, the tannin always and inevitably present in tea, is sufficient to retard digestion, and the amount of tannin taken, and the increased retarding effect produced, depends not upon the length of time the tea is infused, but upon the total quantity of fluid tea drunk. This inhibitory or retarding influence of tea on digestion Sir William Roberts considers to be useful and salutary. Slow digestion does not mean imperfect digestion; and it would appear that the tannin by slowing digestion, and the theine by exhilarating the nervous system, give to tea the extraordinary power of inducing the endurance of fatigue and fasting, of which we all have daily experience, and which makes tea so favourite a beverage with the poor. The afternoon cup of tea, taken with a small amount of bread and butter, will enable a great many hard workers to dispense with luncheon, and to remain without food till a late dinner-hour, without experiencing any discomfort. The inhibitory or slowing influence of tannin is more marked on starch than on albuminates; hence the satisfying character of a good meal of tea and bread, and the probable cause of indigestion and nightmares, and consequent on a "high tea" with cold pie and cakes. A pinch of bicarbonate of soda put into the teapot will destroy the deterrent effect of tea on stomach digestion. (Roberts.)

**Tea has its dangers,** and these are well-known to doctors. If taken in excess, it may produce cardiac disturbances, palpitations, flutterings, a nervous impression of distress and anxiety, and even intermittence of the pulse and sleeplessness, while sometimes it provokes an obstinate form of gastric catarrh. It is said that these effects are due to the tannin contained in the tea, but this assertion is by no means proved. Tannin has but a slight effect on peptic action, and the slowing effect of tea upon stomach digestion is not fully explained by the presence of tannin in tea. (Roberts.) The assertion one hears made that the tannin of tea tans the coats of the stomach into leather is one of those statements which are based more upon a lively imagination than on the data of science. The coats of the stomach do not in any way resemble a hide. They are, moreover, not dead membrane, but living tissue, and the effect of tea on meat fibre is not to harden it, but to cause it to swell. If tea is found to disagree with a person either by over-stimulating the nervous system, or by delaying digestion, the remedy is to take it in smaller quantities or extremely weak, and also not with, but after food. In some forms of dyspepsia, particularly in the hysterical flatulent form, tea should, at all events for a time, be entirely abstained from.

**How to make good Tea.**—The evil effects of tea have been attributed to the methods in vogue of making it. We have seen that the soluble theine is at once dissolved in the hot water, but that the tannin contained in the coarser teas generally used in England, continues to be given off if the tea is left standing on the leaves. Now, this is what almost invariably happens, and the last cup of tea drawn from a pot long standing, and which is said to be "very strong," is strong, not so much in the restorative principle of the theine, but in the astringent tannin which inhibits or slows digestion, and also in the bitter principle which is finally extracted from the leaves. To the habit, customary among the poor, of slowly stewing the tea on the hob, and also to the practice at restaurants and railway stations of continuously boiling it in urns, much of the dyspepsia attributed to tea-drinking is probably due. The reason is, however, not clear. The professional tea-taster allows the boiling water to stand on the tea leaves five minutes and no more; the infusion is then poured off and drunk. If this custom were universally followed we should probably hear fewer complaints about the evil effects of tea-drinking. In order to prevent the tea standing on the tea leaves, various teapots have been invented, by means of which infusion for a certain definite time can be obtained, and the tea leaves are then withdrawn. The best and simplest method is, in my opinion, to have a fine wire basket, in which the measured amount of tea leaves is placed; it is then closed and dropped into the hot water of the teapot for five minutes, after which it is withdrawn. Another method is to have a china strainer under the lid of the teapot, in which the tea leaves are deposited, and the boiling water is poured through the strainer. These teapots are made in Japan, and are imported in large quantities into this country. I have also seen used in Germany a concave perforated metal measure, which is placed at the top of the cup; this is then filled with hot water. When the leaves are sufficiently infused the measure is withdrawn, and the tea leaves are thrown away.

**National Customs in Tea Drinking.**—In England tea is drunk with sugar and milk. Some contend that this custom has been introduced owing to the fact that we drink coarse tea, so strong in tannin, that it is necessary to add sugar and milk to mitigate the astringent flavour. This may be so; but the addition of sugar and milk makes the cup of tea a nourishing food, whereas alone it would only be a stimulant. In Russia, tea is drunk without sugar and milk, but with a slice of lemon added; but there the finest and most costly teas are mostly used. In China and Japan—the home of the tea plant—the drinking of a cup of tea is the invariable accompaniment of all ceremonies. No visit can be paid, no bargain can be struck, no contract can be made, no meal can be taken without a cup of tea. If one is engaged, as is the lot of every traveller, in paying numerous visits both of business and pleasure during the day, it is surprising the number of cups of tea one can consume; and yet tea-dyspepsia and nervousness are unknown in Japan. The cause may be due to the fact that Japanese teas are only slightly roasted and fermented, and that the method of preparation adopted leads to less tannin being dissolved out than by the English method. Every Japanese household, however poor, possesses a large metal tea kettle and a small porcelain or pottery teapot. Into the tiny teapot is placed a small amount of fine green tea. On this is poured not quite boiling water. Without allowing the water to stand on the leaves more than a moment or two, the tea is poured into small porcelain cups, and drunk pure, without any admixture. Tea taken in this way is extraordinarily refreshing. When in Japan, I have sometimes, after being engaged in the fatiguing, incessant, but fascinating occupation of shopping, turned to the saleswoman serving me, and said, *Ocha dozo Okasan*, which means, "Please give me a cup of your honourable tea, good lady," at which request the tiny teapot has been immediately produced with many smiles and bows, and has yielded an astonishing number of small cups of tea, water being continually added from the pretty chased iron kettle. After this "restoration" shopping again became fascinating. There is another kind of tea which is drunk in Japan on the occasion of the unique and solemn Tea Ceremony. This ceremony, which has become a national and tenaciously observed custom, was invented by a great chieftain called Hideyoshi, in the early part of the sixteenth century; with the object of training his turbulent barons to be courteous, self-controlled, and silent. At the Tea Ceremony, the details of which are long, elaborate, and definitely arranged, a fine green tea ground into powder, is brewed in a regulated and ceremonious manner by an official of the household, called the cha-nou. The tea powder is stirred in hot water with a whisk, in an antique bowl. This bowl of tea is handed round to the guests seated on their heels on the matting, and is drunk, tea-dust and all, in solemn silence, the bowl being returned to the cha-nou with forehead bowed to the ground. Thus in this, as in many other things, Japan takes the opposite view to England, but we may, I think, take lessons from Japan to our advantage. and the cup of tea, which in England is notoriously the signal for scandal, is in Japan the motive for a meeting of friends in silence.

